# Pathogenic Variants in Cardiomyopathy Disorder Genes Underlie Pediatric Myocarditis—Further Impact of Heterozygous Immune Disorder Gene Variants?

**DOI:** 10.3390/jcdd9070216

**Published:** 2022-07-05

**Authors:** Franziska Seidel, Kai Thorsten Laser, Karin Klingel, Josephine Dartsch, Simon Theisen, Thomas Pickardt, Manuel Holtgrewe, Anna Gärtner, Felix Berger, Dieter Beule, Hendrik Milting, Stephan Schubert, Sabine Klaassen, Jirko Kühnisch

**Affiliations:** 1 Max Delbrück Center for Molecular Medicine in the Helmholtz Association (MDC), 13125 Berlin, Germany; seidel@dhzb.de (F.S.); josephine.dartsch@mdc-berlin.de (J.D.); simon.theisen@charite.de (S.T.); dieter.beule@bihealth.de (D.B.); 2 Department of Congenital Heart Disease and Pediatric Cardiology, German Heart Center Berlin, 13353 Berlin, Germany; berger@dhzb.de; 3 Experimental and Clinical Research Center, A Cooperation between the Max-Delbrück-Center for Molecular Medicine in the Helmholtz Association and the Charité-Universitätsmedizin Berlin, 13125 Berlin, Germany; 4 DZHK (German Centre for Cardiovascular Research), Partner Site Berlin, 10785 Berlin, Germany; 5 Department of Pediatric Cardiology, Charité-Universitätsmedizin Berlin, 13353 Berlin, Germany; 6 Center for Congenital Heart Disease/Pediatric Cardiology, Heart-and Diabetescenter NRW, University Clinic of Ruhr University Bochum, 32545 Bad Oeynhausen, Germany; tlaser@hdz-nrw.de (K.T.L.); sschubert@hdz-nrw.de (S.S.); 7 Cardiopathology, Institute for Pathology and Neuropathology, University Hospital Tübingen, 72016 Tübingen, Germany; karin.klingel@med.uni-tuebingen.de; 8 National Register for Congenital Heart Defects, 13353 Berlin, Germany; pickardt@kompetenznetz-ahf.de; 9 Core Unit Bioinformatics, Berlin Institute of Health (BIH), 10117 Berlin, Germany; manuel.holtgrewe@bih-charite.de; 10 Core Facility Bioinformatik, Charité-Universitätsmedizin Berlin, Corporate Member of Freie Universität Berlin, Humboldt-Universität zu Berlin, and Berlin Institute of Health, 10117 Berlin, Germany; 11 Erich and Hanna Klessmann-Institute for Cardiovascular Research and Development & Clinic for Thoracic and Cardiovascular Surgery, Heart-and Diabetescenter NRW, University Hospital of the Ruhr University Bochum, 32545 Bad Oeynhausen, Germany; agaertner@hdz-nrw.de (A.G.); hmilting@hdz-nrw.de (H.M.)

**Keywords:** dilated cardiomyopathy, myocarditis, genetic, immune, pathogenic variant

## Abstract

Myocarditis is an inflammatory disease of the heart. Pediatric myocarditis with the dilated cardiomyopathy (DCM) phenotype may be caused by likely pathogenic or pathogenic genetic variants [(L)P] in cardiomyopathy (CMP) genes. Systematic analysis of immune disorder gene defects has not been performed so far. We analyzed 12 patients with biopsy-proven myocarditis and the DCM phenotype together with their parents using whole-exome sequencing (WES). The WES data were filtered for rare pathogenic variants in CMP (*n* = 89) and immune disorder genes (*n* = 631). Twelve children with a median age of 2.9 (1.0–6.8) years had a mean left ventricular ejection fraction of 28% (22–32%) and myocarditis was confirmed by endomyocardial biopsy. Patients with primary immunodeficiency were excluded from the study. Four patients underwent implantation of a ventricular assist device and subsequent heart transplantation. Genetic analysis of the 12 families revealed an (L)P variant in the CMP gene in 8/12 index patients explaining DCM. Screening of recessive immune disorder genes identified a heterozygous (L)P variant in 3/12 index patients. This study supports the genetic impact of CMP genes for pediatric myocarditis with the DCM phenotype. Piloting the idea that additional immune-related genetic defects promote myocarditis suggests that the presence of heterozygous variants in these genes needs further investigation. Altered cilium function might play an additional role in inducing inflammation in the context of CMP.

## 1. Introduction

Myocarditis is an inflammatory entity of the myocardium that may lead to severe heart failure [1]. In the pediatric setting, myocarditis is particularly common in children under two years of age and in adolescents [2,3]. Especially children under two years of age develop the phenotype of dilated cardiomyopathy (DCM) in more than 50% that present with severely impaired left ventricular cardiac function and left ventricular dilatation [4,5]. The clinical courses of these children are severe including increased mortality, the frequent need for mechanical circulatory support (MCS), or heart transplantation (HTx) [5,6,7]. Pediatric DCM, a primary form of cardiomyopathy (CMP) in children, has a peak incidence in the first years of life [8,9]. As there is little difference between the two entities in clinical presentation, differentiation on the basis of clinical data alone is often difficult and usually requires further diagnostic validation with an endomyocardial biopsy (EMB) [10,11]. Most frequently, pediatric myocarditis cases diagnosed with positive EMB are affected by healing/chronic myocarditis and substantial ventricular remodeling with the need for MCS in approximately 20% and HTx in 10% of cases [6,12].

Myocarditis arises mostly from infectious causes, less frequently from (auto)immunological predisposition, or drug toxicity [13]. Viral infection with parvovirus B19 (PVB19), enteroviruses, and human herpes virus 6 (HHV-6) are among the classic pathogens of myocarditis [14]. Mechanistically, myocarditis involves innate and adaptive immune system components that become activated after virus entry via their common receptors (e.g., coxsackie–adenoviral receptor (CAR), decay-accelerating factor (DAF)) and activation of the innate immune response via toll-like receptors (e.g., TLR3, TLR4). Consequently, natural killer cells and macrophages become recruited to the myocardium, their activation releases IFN-α/β, and initiates T-/B-lymphocyte proliferation and activation [1,15,16]. Subsequently, T-lymphocytes target viral proteins but also expose mimicry mechanisms towards myocardial proteins such as the myosin heavy chain [16,17]. This suggests that myocarditis is an intertwined pathology of immune defense against external pathogens and autoimmune response to myocardial antigens [1,16]. The frequent association of DCM with inflammation implicates a critical predisposition of the myocardium [12,18,19,20].

Recently, we showed that pediatric myocarditis with the DCM phenotype is due to pathogenic or likely pathogenic genetic variants [(L)P] in CMP disease genes [12]. In this study, we could discriminate between a clinical group presenting myocarditis with the DCM phenotype (MYC-DCM) and myocarditis without the DCM phenotype (MYC-nonDCM). Clinically, the MYC-DCM group had much worse outcomes compared to the MYC-nonDCM group. Most children of the MYC-nonDCM group recovered after myocarditis. However, patients of the MYC-DCM group often required MCS or HTx and carry more frequently genetic (L)P variants. This suggests that a proportion of pediatric patients with myocarditis have an underlying primary, genetically determined DCM.

The potential role of genetic factors in immune disorder genes was assessed in pediatric patients with acute myocarditis [21]. However, this study could not identify the enrichment of rare heterozygous, homozygous genetic variants in *TLR3* and IFN-α/β associated genes. Homozygous variants in CMP disease genes such as desmoplakin (*DSP*) and troponin I3 (*TNNI3*) were identified in pediatric patients with myocarditis [21]. The critical role of genetic (L)P variants in *DSP* and *TNNI3* was replicated in several studies involving children or adults with myocarditis [12,18,19,22,23,24]. Another recent study identified protein-truncating variants in titin (*TTN*-tv), filamin C (*FLNC*), and *DSP*, which are CMP disease-related genes*,* in a cohort of adult lymphocytic myocarditis cases [25]. A seminal study linked an immunoregulatory locus comprising the major histocompatibility complex, class II, DR beta 4 (*HLA-DRB4*) with idiopathic DCM [26]. Single-case or small cohort studies identified Interleukin 12 receptor subunit beta 1 (*IL12RB1*) variants and the TLR3 p.Pro554Ser variant as genetic susceptibility factors for myocarditis [27,28]. Altogether, this suggests that mutation of CMP genes is associated with myocarditis, and genetic defects of immune system components are heterogeneous. The impact of hetero-, hemi-, or homozygous genetic variants in a broader spectrum of known immune disease genes was not systematically explored in mono- or oligogenic traits so far. Here, we test the hypothesis that patients with severe DCM and biopsy-proven myocarditis carry genetic variants in CMP and immune disease genes utilizing a family-based genetic approach.

## 2. Material and Methods

### 2.1. Study Population

Clinical data from patients below 18 years of age with suspected myocarditis were extracted from medical records at the Pediatric Cardiology Departments of the Charité-Universitätsmedizin Berlin, the German Heart Center Berlin, and the Heart- and Diabetescenter NRW, Bad Oeynhausen, Germany between January 2010 and April 2021. Starting in 2013, six patients were also enrolled in MYKKE, a prospective multicenter registry for suspected myocarditis (clinical trial identifier: NCT02590341; accessed on 1 April 2021). The online database of the MYKKE registry includes patients’ clinical data and is hosted by the Competence Network for Congenital Heart Defects, Germany [4]. This study used a family approach including genetic analysis of the index patient and the respective parents.

The inclusion criteria of index patients were the following: Biopsy-proven myocarditis and present DCM phenotype at admission, as previously described before [12]. Patients with structural congenital heart defects, syndromic disorders, situs inversus, or metabolic, mitochondrial, or neuromuscular disease were excluded from the study. Clinical and diagnostic assessments including laboratory parameters and cardiac imaging were performed routinely. The follow-up for the occurrence of major adverse cardiac events such as malign arrhythmia, MCS, HTx, and/or all-cause death started with the date of admission. The study was conducted according to the guidelines of the Declaration of Helsinki, approved by the institutional ethics committee (Charité-Universitätsmedizin Berlin, ID EA2/083/13, EA2/131/10, EA2/074/13; University Clinic of Ruhr University Bochum, Bad Oeynhausen for MYKKE 65/2013 and Biobank 21/2013). All parents/guardians of patients <18 years gave written informed consent.

### 2.2. Statistical Analysis for Clinical Data

Categorical variables were summarized by frequencies and percentages. For continuous measures, data were presented as median values with the interquartile range (IQR). Pearson’s chi-square test and Fisher’s exact test were used to compare dichotomous variables. For the comparison of independent groups, the Mann–Whitney U and Kruskal–Wallis tests were applied. A probability value of <0.05 was considered statistically significant. Data were analyzed using IBM Corp. Released 2017, IBM SPSS Statistics for Windows, Version 25.0. (Armonk, NY, USA).

### 2.3. Analysis of Endomyocardial Biopsies

Endomyocardial biopsies (EMB) were taken according to the clinical routine from the left, right, or both ventricles as a diagnostic approach or transmural specimen during VAD implantation from all patients (*n* = 12). All biopsies were analyzed histopathologically and immunohistologically as previously described and by quantitative polymerase chain reaction (qPCR) for myocardial detection of viral RNA/DNA by one specialized center for Cardiopathology (Institute for Pathology and Neuropathology, University Hospital Tübingen, Tübingen, Germany) [14,29,30]. Histological analysis followed the Dallas criteria as the gold standard for the evaluation of myocarditis and was completed by different immunohistochemical staining [30]. The diagnosis of myocarditis was confirmed according to the established criteria and categorized in accordance with the WHO definition [31,32].

### 2.4. Next-Generation Sequencing (NGS) and Variant Calling

For NGS analysis, DNA was isolated from peripheral blood (Macherey-Nagel, Germany) and quality checked with Qubit (Invitrogen, Carlsbad, CA, USA) and Bioanalyzer (Agilent Technologies, Santa Clara, CA, USA.) [33]. Whole-exome sequencing (WES) occurred at Genewiz (Azenta Life Sciences, Leipzig, Germany). The genomic DNA was quantified using the Qubit 4.0 Fluorometer and qualified using the Agilent 5300 Fragment Analyzer. Enrichment probes were designed against the region of interest and synthesized through Twist Comprehensive Exome kit Biosciences (South San Francisco, CA, USA). Library preparation was performed according to the manufacturer’s guidelines. Briefly, the genomic DNA was fragmented enzymatically according to the manufacturer’s instructions. Fragmented DNA was cleaned up and end-repaired, as well as adenylated at the 3’ends. Adapters were ligated to the DNA fragments, and adapter-ligated DNA fragments were enriched with limited-cycle PCR. Adapter-ligated DNA fragments were validated using the Agilent Fragment Analyzer (Agilent Technologies, Santa Clara, CA, USA), and quantified using the Qubit 2.0 Fluorometer. Adapter-ligated DNA fragments were hybridized with biotinylated baits. The hybrid DNAs were captured by streptavidin-coated binding beads. After extensive washing, the captured DNAs were amplified and indexed with Illumina indexing primers. Post-captured DNA libraries were validated using the 5300 Fragment Analyzer (Agilent, Santa Clara, CA, USA) and quantified using the Qubit 2.0 Fluorometer. Illumina reagents and kits for DNA library sequencing cluster generation and sequencing were used for enrichment DNA sequencing. Post-captured libraries have been multiplexed on a flow-cell and loaded on the Illumina NovaSeq 6000 instrument according to the manufacturer’s instructions (Illumina, San Diego, CA, USA). The samples were sequenced using a 2 × 150 paired-end (PE) configuration. Image analysis and base calling were conducted by NovaSeq Control Software on the NovaSeq instrument. Raw sequencing data (bcl files) generated from Illumina NovaSeq were converted into fastq files and de-multiplexed using Illumina’s bcl2fastq software.

Alignment of WES raw data sets and variant calling was performed using the GRCh37 (hs37d5.fa) reference genome [33]. Briefly, the demultiplexing of libraries occurred with bcl2fastq v2.17.1.14, the reads were aligned using BWA-MEM v0.7.15 (Wellcome Trust Sanger Institute, Cambridge, UK) to the reference GRCh37 (hs37d5.fa), separate read groups were assigned for all reads from one lane, and duplicates were masked using Samblaster v0.1.24 (Ira Hall Lab, University of Virgina, VA, USA). FastQC was used for standard quality control. The variants were then called using GATK UnifiedGenotyper v3.7 (Broad Institute, Cambridge, MA, USA). The called variants were evaluated with Varfish using a minor allele frequency (MAF) < 0.0001 and mutation specification [34]. We used the Genome Aggregation Database (gnomAD v.2.) as a genetic reference database for allele frequencies in a control cohort (https://gnomad.broadinstitute.org/; accessed on 1 April 2021, Broad Institute, Cambridge, MA, USA) [35]. We evaluated 89 validated CMP disease genes [33,36] and 631 validated immune disease genes (https://blueprintgenetics.com; accessed on 1 April 2021, Blueprint Genetics, Espoo, FIN) [37,38,39].

#### 2.4.1. Gene List of 89 CMP Disease Genes

Variants in the following genes were bioinformatically evaluated and classified: *ABCC9, ACTA1, ACTC1, ACTN2, **ALMS1**, ANKRD1, BAG3, BRAF, CALR3, CAV3, CBL, **COX15**, CRYAB, CSRP3, DES, **DMD** (*XL*), **DNAJC19, DOLK**, DSC2, DSG2, DSP, DTNA, **EMD** (*XL*), EYA4, FBN1, **FHL1** (*XL*), FHL2, **FKRP, FKTN, FXN, GAA, GATAD1**, GLA (*XL*), **HADHA**, HCN4, **HFE**, HRAS, HSPB8, JPH2, JUP, KRAS, **LAMA2**, LAMA4, LAMP2 (*XL*), LDB3, LMNA, MAP2K1, MAP2K2, MIB1, MYBPC3, MYH6, MYH7, MYL2, MYL3, MYLK2, MYOZ2, MYPN, NEXN, NKX2-5, NRAS, PDLIM3, PKP2, PLN, PRDM16, PRKAG2, PTPN11, RAF1, RBM20, RYR2, SCN5A, **SCO2**, **SDHA**, **SGCB**, SGCD, **SGCG**, SHOC2, SOS1, **TAFAZZIN** (*XL*), TBX20, TCAP, TGFB3, TMEM43, TNNC1, TNNI3, TNNT2, TPM1, TTN, TTR, VCL*. Genes given in regular format were considered disease-causing only with autosomal dominant (AD) inheritance. Genes in **bold** were considered disease-causing only with autosomal recessive (AR) inheritance.

#### 2.4.2. Gene List of 631 Immune Disease Genes

Variants in the following genes were bioinformatically evaluated and classified: ***ABCB7** (*XL*), **ABCG5**, **ABCG8**, **ACD**, **ACP5**, ACTB, ACTG1, ACTN1, **ADA**, **ADAM17**, **ADAMTS13**, **ADAMTS3**, **ADAR**, **ADIPOQ**, **ADIPOR1**, **ADIPOR2**, **AICDA**, **AIRE**, **AK2**, **ALAS2** (*XL*), **ALPI**, ANKRD11, ANKRD26, AP1S3, **AP3B1**, **AP3D1**, **APOL1**, **ARHGEF1**, **ARMC4**, **ARPC1B**, **ATM**, **ATP6AP1** (*XL*), **ATR**, **ATRX** (*XL*), **B2M**, BACH2, **BCL10**, BCL11B, **BCO1**, **BLM**, **BLNK**, **BLOC1S3**, **BLOC1S6**, BRAF, BRCA1, **BRCA2**, **BRIP1**, **BTK** (*XL*), **C1QA**, **C1QB**, **C1QBP**, **C1QC**, **C1R**, **C1S**, **C2**, **C3**, **C3AR1**, **C4BPA**, **C4BPB**, **C5**, **C5AR1**, **C5AR2**, **C6**, **C7**, **C8A**, **C8B**, **C8G**, **C9**, **CARD11**, CARD14, **CARD9**, CASP10, **CASP8**, CBL, **CCBE1**, **CCDC103**, **CCDC114**, **CCDC39**, **CCDC40**, **CCDC65**, CCNK, **CCNO**, **CD19**, **CD247**, **CD27**, **CD3D**, **CD3E**, **CD3G**, **CD40**, **CD40LG** (*XL*), **CD46**, **CD55**, **CD59**, **CD70**, **CD79A**, **CD79B**, **CD81**, **CD8A**, **CD93**, **CDAN1**, CDC42, **CDCA7**, **CDK9**, CDKN2A, CEBPA, **CEBPE**, **CENPF**, **CFB**, **CFD**, **CFH**, **CFHR5**, **CFI**, **CFP** (XL), **CFTR**, CHD7, **CHEK2**, **CIB1**, **CIITA**, **CLCN7**, **CLEC7A**, **CLPB**, **CLU**, **COG6**, **COLEC11**, COPA, **CORO1A**, **CPT2**, **CR2**, CREBBP, **CRP**, **CSF2RA** (*XL*), **CSF2RB**, CSF3R, **CTC1**, CTLA4, CTNNBL1, **CTPS1**, **CTSC**, **CXCR2**, CXCR4, **CYBA**, **CYBB** (*XL*), CYCS, **CYP27A1**, **DBR1**, DCLRE1B, **DCLRE1C**, **DDX11**, DDX41, DDX58, **DEF6**, **DGAT1**, **DGKE**, **DHFR**, **DKC1 (***XL***)**, **DNAAF1**, **DNAAF2**, **DNAAF3**, **DNAAF5**, **DNAH1**, **DNAH11**, **DNAH5**, **DNAH9**, **DNAI1**, **DNAI2**, **DNAJC21**, **DNAL1**, **DNASE1L3**, **DNASE2**, **DNMT3B**, **DOCK2**, **DOCK8**, **DRC1**, **DTNBP1**, **EFL1**, **EIF2AK3**, ELANE, EP300, **EPCAM**, **EPG5**, **EPO**, **ERCC2**, **ERCC3**, **ERCC4**, **ERCC6L2**, ETV6, **EXTL3**, FAAP100, FAAP24, **FADD**, **FANCA**, **FANCB** (*XL*), **FANCC**, **FANCD2**, **FANCE**, **FANCF**, **FANCG**, **FANCI**, **FANCL**, **FANCM**, **FAS**, FASLG, **FAT4**, **FCGR3A**, **FCHO1**, **FCN1**, **FCN2**, **FCN3**, **FERMT1**, **FERMT3**, **FLG**, FLI1, **FLNA** (*XL*), **FOXN1**, **FOXP3** (XR), FPR1, **G6PC3**, **G6PD** (*XL*), **GAS2L2**, **GAS8**, **GATA1** (*XL*), GATA2, **GBA**, GFI1, GFI1B, **GINS1**, **GLRX5**, **GNE**, **GP1BA**, **GP1BB**, **GP9**, **GTF2H5**, **GUCY2C**, **HAVCR2**, **HAX1**, **HELLS**, **HMOX1**, HNRNPK, HOXA11, **HPS1**, **HPS3**, **HPS4**, **HPS5**, **HPS6**, HRAS, **HSPA9**, **HYDIN**, **HYOU1**, **ICOS**, ICOSLG, **IFIH1**, IFNAR1, **IFNAR2**, **IFNGR1**, **IFNGR2**, **IGLL1**, **IKBKB**, IKZF1, IL10, **IL10RA**, **IL10RB**, **IL12B**, **IL12RB1**, IL12RB2, IL17F, **IL17RA, IL17RC**, **IL18BP**, **IL1RN**, **IL21**, **IL21R**, **IL23R**, **IL2RA**, **IL2RB**, **IL2RG** (*XL*), **IL36RN**, **IL6R**, **IL6ST**, **IL7R**, INO80, **INVS**, IRAK1, IRAK4, IRF2BP2, IRF3, IRF4, **IRF7**, **IRF8**, **IRF9**, **ISG15**, **ITCH**, **ITGA2**, **ITGA2B**, **ITGB2**, **ITGB3**, **ITK**, **JAGN1**, **JAK1**, JAK2, **JAK3**, KDM1A, **KDM6A** (*XL*), KIF23, KLF1, KMT2A, KMT2D, KRAS, **LAMTOR2**, **LAT**, **LCK**, **LCT**, **LIG1**, **LIG4**, **LIPA**, **LPIN2**, **LRBA**, **LRRC6**, LRRC8A, **LYST**, **LZTR1**, **MAD2L2**, **MAGT1** (*XL*), **MALT1**, **MAN2B1**, **MANBA**, MAP2K1, MAP2K2, **MAP3K14**, MAP3K8, **MASP1**, **MASP2**, MASTL, **MAT2A**, MBL2, **MCIDAS**, **MCM4**, MECOM, **MEFV**, **MLH1**, **MLPH**, **MOGS**, **MPL**, **MPO**, MRAS, **MS4A1**, **MSH2**, **MSH6**, **MSN** (*XL*), **MTHFD1**, **MVK**, **MYD88**, MYH9, **MYO5A**, **MYO5B**, **MYSM1**, NAF1, **NBAS**, **NBEAL2**, **NBN**, **NCF1**, **NCF2**, **NCF4**, **NCKAP1L**, NCSTN, **NEUROG3**, NF1, NFAT5, NFE2L2, NFIL3, NFKB1, NFKB2, NFKBIA, **NHEJ1**, **NHP2**, NLRC4, **NLRP1**, NLRP12, NLRP3, **NME8**, NOD2, **NOP10**, NRAS, **NSMCE2**, **NSMCE3**, **NUP214**, **OAS1**, **OFD1** (*XL*), **ORAI1**, **OSTM1**, **OTUD6B**, **OTULIN**, **PALB2**, **PARN**, PAX5, **PEPD**, **PGM3**, **PIGA** (*XL*), **PIH1D3** (*XL*), PIK3CD, **PIK3R1**, PLCG2, **PLEKHM1**, **PLG**, **PMM2**, **PMS2**, **PNP**, **POLA1** (*XL*), **POLD1**, **POLD2**, **POLE**, **POLE2**, **POLR3A**, POLR3C, POLR3F, **POMP**, POT1, PPP1CB, **PRF1**, **PRG4**, **PRKACG**, **PRKCD**, **PRKDC**, PSEN1, PSENEN, **PSMB8**, **PSMG2**, PSTPIP1, PTEN, PTPN11, **PTPRC**, **PTX3**, **PUS1**, **RAB27A**, RAC2, **RAD50**, RAD51, **RAD51C**, RAF1, **RAG1**, **RAG2**, RANBP2, RAP1A, RAP1B, RASA2, **RASGRP1**, **RBCK1**, **RBM8A**, **RECQL4**, **REL**, RELA, **RELB**, **RFWD3**, **RFX5**, **RFXANK**, **RFXAP**, **RHOH**, **RIPK1**, RIT1, **RMRP**, **RNASEH2A**, **RNASEH2B**, **RNASEH2C**, **RNF168**, **RNF31**, **RNU4ATAC**, **RORC**, **RPGR** (*XL*), **RPL10** (*XL*), RPL11, RPL15, RPL18, RPL19, RPL26, RPL27, RPL31, RPL35A, RPL36, RPL5, RPL9, RPS10, RPS14, RPS15, RPS15A, RPS19, RPS24, RPS26, RPS27, RPS27A, RPS28, RPS29, RPS7, RPSA, **RRAS**, **RSPH1**, **RSPH3**, **RSPH4A**, **RSPH9**, **RTEL1**, RUNX1, **SAMD9**, SAMD9L, **SAMHD1**, **SAR1B**, **SBDS**, **SBF2**, **SEC23B**, SEC61A1, SEMA3E, **SERPING1**, **SH2D1A** (*XL*), SH3BP2, **SH3KBP1** (*XL*), SHOC2, **SI**, **SKIV2L**, SLC10A2, **SLC19A2**, **SLC25A38**, **SLC26A3**, **SLC29A3**, **SLC35A1**, **SLC35C1**, **SLC37A4**, **SLC39A4**, **SLC39A7**, **SLC46A1**, **SLC5A1**, **SLC7A7**, **SLC9A3**, **SLFN14**, **SLX4**, **SMARCAL1**, **SMARCD2**, **SNX10**, SOS1, SOS2, **SP110**, **SPAG1**, **SPINK5**, **SPINT2**, **SPPL2A**, SPRED1, SRC, SRP54, SRP72, **STAT1**, **STAT2**, STAT3, **STAT5B**, **STIM1**, **STK36**, **STK4**, **STX11**, **STX3**, **STXBP2**, STXBP3, **TAP1**, **TAP2**, **TAPBP**, **TASP1**, **TAFAZZIN** (*XL*), TBK1, TBX1, TCF3, **TCIRG1**, **TCN2**, TERC, TERF2, TERF2IP, **TERT**, **TFRC**, TGFB1, TGFBR1, TGFBR2, THBD, THPO, THRA, **THRB**, **TICAM1**, TINF2, TIRAP, **TLR3**, **TMC6**, **TMC8**, TMEM173, TNFAIP3, **TNFRSF11A**, **TNFRSF13B**, **TNFRSF13C**, TNFRSF1A, **TNFRSF4**, TNFRSF9, **TNFSF11**, TNFSF12, TOP2B, TP53, **TPP2**, **TRAC**, TRADD, TRAF3, **TRAF3IP2**, **TREX1**, TRIM22, **TRNT1**, **TSR2** (*XL*), **TTC37**, **TTC7A**, TUBB1, **TYK2**, **UBE2T**, UNC119, **UNC13D**, **UNC93B1**, **UNG**, **USB1**, **USP18**, **VPS13B**, **VPS45**, **VSIG4** (*XL*), **VTN**, **WAS** (*XL*), **WDR1**, **WIPF1**, **WRAP53**, **XIAP** (*XL*), **XRCC2**, **ZAP70**, **ZBTB24**, ZCCHC8, **ZMYND10**, **ZNF341***. Genes given in regular format were considered disease-causing only with AD inheritance. Genes in **bold** were considered disease-causing only with AR inheritance. Genes in underlined **bold** were considered disease-causing with AD or AR inheritance. Genes with X-linked (XL) inheritance are indicated.

### 2.5. Genetic Analysis and Variant Classification

All filtered genetic variants were classified as pathogenic (P), likely pathogenic (LP), or a variant of uncertain significance (VUS) according to the guidelines of the American College of Medical Genetics and Genomics (ACMG) [40,41]. The MAF for variant filtering was <0.0001. Novel (L)P genetic variants will be deposited in the National Center for Biotechnology (NCBI) database ClinVar, available at: https://www.ncbi.nlm.nih.gov/clinvar/submitters/506935/ (accessed on 1 April 2021, NCBI, Bethesda, MD, USA). The filter criteria for the 89 CMP disease genes included the following variants: Missense, protein length changing, splice effects (until +4 donor, until -4 acceptor site) in either genetic status (hetero-, homo-, hemizygous). The filter criteria for the 631 immune disease genes included the following variants: Missense only when annotated in ClinVar as (L)P variant or appear homo-/hemizygous, protein length changing, splice effects (until +4 donor, until -4 acceptor site) in either genetic status (hetero-, homo-, hemizygous). The following variant types were assessed: Single nucleotide variants (SNV), insertion/deletion (indel), and multi-nucleotide variants (MNV). Variant annotation occurred according to Ensembl (http://www.ensembl.org/index.html; accessed on 1 April 2021, European Bioinformatics Institute (EMBL-EBI), Hinxton Cambridge, UK).

Variant classification was performed according to ACMG guidelines [41]. The term PVS1 was applied when loss of function (LOF), truncating variants are a proven CMP disease mechanism. PVS1 was applied for *DSP*, *BAG3*, and *TNNI3* only. For *TTN*-tv, we did not apply PVS1. The ACMG terms PM5 or PS1 were activated when published LP/P variants occurred at the same amino acid position or amino acid exchange, respectively. PS3 was applied when database evidence, e.g., PubMed (https://pubmed.ncbi.nlm.nih.gov; accessed on 1 April 2021, NCBI, Bethesda, MD, USA), Ensembl (http://www.ensembl.org/index.html, accessed on 1 April 2021), UniProt (https://www.uniprot.org/; accessed on 1 April 2021, EMBL-EBI, Hinxton Cambridge, UK), or ClinVar (https://www.ncbi.nlm.nih.gov/clinvar/; accessed on 1 April 2021, NCBI, Bethesda, MD, USA), provided a clear pathological impact of the variant. PM1 was used when variants in proximity affected the functional domain or LP/P variants accumulated close to the analyzed variant. The ACMG term PM2 was activated at a gnomAD MAF < 0.0001. PM4 was applied for protein-length-changing variants due to coding sequence changes. PM6 was activated when a *de novo* variant was detected without confirmation of paternity and maternity. PP3 was activated when in silico prediction tools, e.g., MT2 (http://www.mutationtaster.org/; accessed on 1 April 2021, Bioinformatics and Translational Genetics, Berlin Institute of Health (BIH), Berlin, Germany) or Provean (http://provean.jcvi.org/index.php; accessed on 1 April 2021, J. Craig Venter Institute, La Jolla, CA, USA), provided a negative impact of the variant. For classification, the National Center for Biotechnology (NCBI) database ClinVar (https://www.ncbi.nlm.nih.gov/clinvar/; accessed on 1 April 2021, NCBI, Bethesda, MD, USA) was applied. Missense variants in *TTN* were not evaluated. The impact of *TTN* length-changing variants was validated according to the splice pattern displayed at cardiodb (https://www.cardiodb.org/titin; accessed on 1 April 2021, Medical Research Council (MRC), Imperial College London, London, UK) [42]. Genetic variants detected in index patients were traced in first-degree family members to determine *de novo* mutation or segregation. The Combined Annotation-Dependent Depletion (CADD) score was calculated for all variants (https://cadd.gs.washington.edu/score; accessed on 1 April 2021, BIH, Berlin, Germany) [43]. The CADD score is a quantitative prioritization approach for genetic variants implementing multiple computational predictions including conservation metrics, functional genomic information, transcript information, and protein scores. The CADD score ranges from 1 to 99, and higher values indicate a more deleterious impact of a genetic alteration. The threshold > 10–20 indicates deleterious substitutions.

## 3. Results

### 3.1. Clinical Characterization

We enrolled 12 families including the pediatric index patient (*n* = 12) and both parents (*n* = 24). The index patients had a median age of 1.6 (0.8–8.0) years, including five males and seven females (Table 1). All children presented with a DCM phenotype. The mean left ventricular ejection fraction (LVEF) was 23% (21–30%) and the Z-Score of the left ventricular internal dimension at end-diastole (LVIDd) 6.6 (5.4–8.0). All had heart failure symptoms, received heart failure medication, and needed inotropic support. As immunological events, in seven patients, a viral respiratory infection within six weeks before admission was determined. One patient received vaccination against tetanus, diphtheria, whooping cough, haemophilus influenza b, polio, and hepatitis B one week before admission. Another patient was a small-gestational-age premature infant (index Family #5) and one was diagnosed later in life with Morbus Crohn (index Family #9). Severe, underlying immune disorders were excluded in all patients and parents during in-depth clinical assessment. EMB diagnosis revealed myocarditis-associated inflammation in all patients: Acute myocarditis *n* = 1, chronic/healing myocarditis *n* = 8, and unspecific macrophage dominated inflammation *n* = 3. Myocardial virus was detected in 6 out of 12 patients (PVB19, *n* = 2; HHV-6, *n* = 4). Most of these patients had low viral loads (*n* = 5). Eleven patients underwent implantation of a ventricular assist device (VAD) and nine patients subsequently received HTx; none died. One patient could be weaned from VAD. This patient had moderate viral myocardial PVB19 levels.

### 3.2. Identification of Genetic CMP Disease Variants

**Figure 1:**
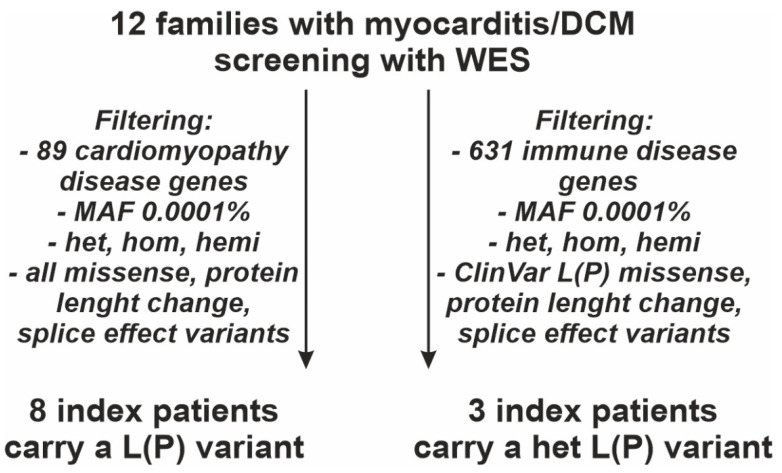
Study flow chart of the genetic evaluation after whole-exome sequencing (WES), including only patients with biopsy-proven myocarditis and phenotype of dilated cardiomyopathy (DCM). Cardiomyopathy (CMP) disease genes and immune disease genes were filtered with minor allele frequency (MAF) of 10^−4^ for pathogenic and likely pathogenic, (L)P, variants. Filtering for cardiomyopathy and immune disease genes included all heterozygous (het), homozygous (hom), and hemizygous (hemi) variants. Analysis of cardiomyopathy disease genes included all missense, protein length changing, and splice effect variants. Analysis of immune disease genes included all protein length changing and splice effect variants, but only missense (L)P variants previously annotated in ClinVar.

**Figure 2:**
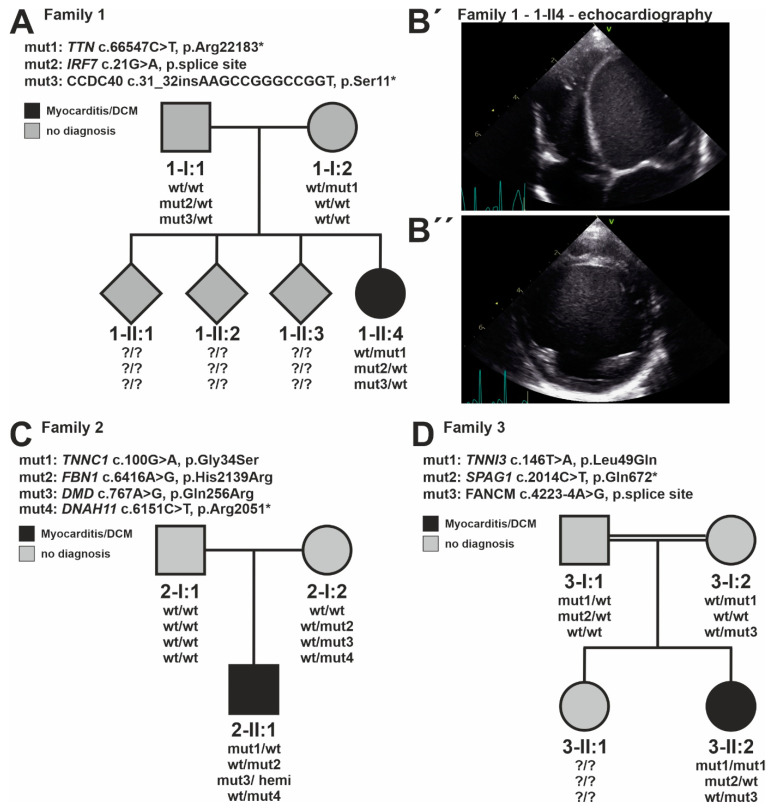
Clinical and genetic evaluation of three selected families with CMP and myocarditis. (**A**) Pedigree of family #1 with the index patient 1-II:4 carrying a heterozygous titin truncating variant (*TTN*-tv), a splice variant in interferon regulatory factor 7 (*IRF7*), and a protein-truncating variant in coiled-coil domain containing 40 (*CCDC40*). The *IRF7* and *CCDC40* alleles are inherited from the father, while the *TTN*-tv allele is of maternal origin. (**B′**) Echocardiographic images of 4-chamber view and (**B″**) midventricular and short axis of individual 1-II:4 reveal severe left ventricular dilatation. (**C**) Pedigree of family #2 with the index patient 2-II:1 carrying a heterozygous *de novo* variant in troponin C1, slow skeletal and cardiac type (*TNNC1*), missense variants in fibrillin 1 (*FBN1*), as well as dystrophin (*DMD*), and a stop gain variant in dynein axonemal heavy chain 11 (*DNAH11*). All alleles are inherited from the mother. The *DMD* missense allele appears hemizygously. (**D**) Pedigree of family #3 with the index patient 3-II:2 exposes a homozygous missense variant in troponin I3, cardiac type (*TNNI3*), a stop gain variant in sperm-associated antigen 1 (*SPAG1*), and a splice variant in FA complementation group M (*FANCM*). The *SPAG1* allele is inherited from the mother. The parents are consanguine.

Genetic analysis of the 12 families revealed an (L)P variant in a CMP gene in 8/12 index patients related to DCM (Figure 1, Table 2). Assessing genetic variants in 89 CMP disease genes yielded one (L)P variant in eight patients, at least one VUS in three patients, and no genetic CMP variant was found in one patient. In the index patient of family #10, the compound heterozygous genotype of two VUS missense variants in fukutin (*FKTN*), inherited in trans from the mother and father, likely explains the clinical phenotype composed of skeletal myopathy and DCM [44]. From these eight (L)P variants, six were missense and two emerged as stop gain variants. For instance, the truncating variant *TTN* p.Y8199* is annotated as (L)P variant (VCV000223326). All these (L)P variants have a CADD value > 23. Three (L)P variants appeared *de novo*, and one was homozygous (Figure 2). Four (L)P variants were novel and have not been reported in ClinVar so far. These genetic variants were found in troponin C1, slow skeletal and cardiac type (*TNNC1*) p.G34S (*de novo*, index patient of family #2), *TNNI3* p.E182Q (*de novo*, index patient of family #7), *TNNI3* p.L49Q (hom, family #3), and *TTN* p.Y8199 * (stop gain, family #9). The high yield of CMP causative (L)P variants reflects the severe clinical stage of the analyzed pediatric cohort.

### 3.3. Identification of Immune Disease Gene Variants

Genetic analysis of the 12 families revealed an (L)P variant in an immune disorder gene in 3/12 index patients. More specifically, genetic analysis of 631 immune disease genes yielded at least a VUS in nine patients and no genetic immune variant in three patients (Table 2). Overall, 15 variants were detected, including 3 (L)P variants and 12 VUS. These comprise six stop gain (truncation) variants, five splice region variants, two missense variants, and one in-frame deletion variant. All three (L)P variants were annotated in ClinVar as (L)P, are in a heterozygous state, have CADD values >30, and represent stop gain variants: Dynein axonemal heavy chain 11 (*DNAH11*) p.R2051*, FA complementation group C (*FANCC*) p.R548*, and sperm-associated antigen 1 (*SPAG1*) p.Q672* (Figure 2). Of note, these variants were associated with recessive inheritance for Fanconi anemia, complementation group C (*FANCC*), primary ciliary dyskinesis 7 (*DNAH11*), and primary ciliary dyskinesis 28 (*SPAG1*) so far. Within the 12 families, three variants were found in *FANCC* and FA complementation group M (*FANCM*) that were associated with Fanconi anemia and spermatogenic failure, respectively. Considering the molecular function of the immune disease genes, we observe an accumulation of variants in genes directly or indirectly associated with ciliary transport: Coiled-coil domain containing 40 (*CCDC40*), dynein axonemal heavy chain 9 (*DNAH9*), *DNAH11*, PIH1 domain-containing protein 3 (*PIH1D3*), and *SPAG1*. Further analysis excluded another missense variant in trans of the same gene for all filtered 15 immune gene variants. This dismisses compound heterozygosity for these genes. The CMP variant *TTN* p.Y8199* was maternally inherited in the index patient of family #1, while both immune-related variants originate from the father (Figure 2). Whether such an allelic combination is relevant for the development of myocarditis with the DCM phenotype should be tested in larger cohorts.

## 4. Discussion

Using a family-based approach, this study provides further evidence that pediatric myocarditis with the DCM phenotype can be genetically caused by the mutation of established CMP disease genes. The spectrum of (L)P variants found in this study comprises missense variants in *MYH7*, *TNNC1*, *TNNI3*, ryanodine receptor 2 (*RYR2*) but also *TTN*-tv, which is expected in a DCM cohort [36,45]. Thus, (L)P variants in CMP disease genes may underlie pediatric myocarditis, likely by affecting the tissue and cellular integrity of the myocardium. This study yielded an (L)P variant in a CMP gene in 8/12 patients reflecting the severe clinical disease course of the patients in the majority requiring VAD and HTx. All children with (L)P CMP variants presented with symptoms of severe heart failure, which is known to trigger further inflammation [46]. The elevated troponin levels indicate myocardial damage and might also trigger ongoing immune-induced damage to cardiomyocytes.

We piloted the idea that additional immune-related genetic defects promote inflammation. Heterozygous truncation variants with high CADD scores in immune-related genes were detected in 6/12 index patients and may have led to partial immunological dysfunction. Of note, this study did not identify genetic alterations in a single gene or isolated functional complex, indicating that myocarditis may be associated with several immune pathway defects. Three variants in immune genes were categorized in ClinVar as (L)P in association with the AR inheritance mode for Fanconi anemia, complementation group C (*FANCC*), primary ciliary dyskinesis 7 (*DNAH11*), and primary ciliary dyskinesis 28 (*SPAG1*). Due to limitations of the ACMG guidelines and ClinVar annotations, these (L)P variants in recessive genes cannot be classified as pathogenic in the heterozygous state. Importantly, these patients had no obvious signs of Fanconi anemia or primary ciliary dyskinesis. Interpretation of these variants is hampered by a lack of knowledge about mild clinical signs in the heterozygous genotype. The heterozygous variant *FANCC*, c.1642C > T, p.R548* is discussed as a low penetrance risk allele for breast cancer [47]. Of note, we found no genetic alteration in molecular mechanisms such as TLR, IFN-α/β, or interleukin signaling previously associated with myocarditis. Low penetrant immune phenotypes might induce clinical problems only after special triggers such as DCM with the phenotype of myocarditis. However, the limited understanding of monoallelic deactivation for most immune disease genes hinders further genetic interpretation. This limitation will be overcome by future functional and clinical characterization of these genes and genetic variants.

Considering the immune-related genes, this study found genetic variants in genes such as *CCDC40, DNAH9*, *DNAH11*, *PIH1D3*, and *SPAG1* directly or indirectly associated with ciliary transport. The primary cilium is a specialized, non-motile cellular extrusion implicated in sensing and cellular signaling required for many developmental processes and cellular functions [48]. More importantly, *DNAH9, DNAH11*, *PIH1D3*, and *SPAG1* were classified as primary ciliary dyskinesia (PCD) disease genes typically with AR inheritance; *PIH1D3* follows X-linked recessive (XLR) inheritance [49]. Most frequently, PCD is linked to left–right axis abnormalities, defects in cardiac development, conductive hearing problems, subfertility, and respiratory problems associated with respiratory infections; typically, with biallelic gene deactivation. Moderate, subclinical phenotypes after the mutation of *CCDC40, DNAH9*, *DNAH11*, *PIH1D3*, and *SPAG1* have not been described. In immune cells, the immunological synapse serves as a molecular interface between the antigen-presenting cell and the interacting, activated lymphocyte [50]. From a molecular perspective, the immunological synapse is a specialized cilium structure sharing, for instance, similarities in centrosomal positioning at the plasma membrane, actin organization, and molecules facilitating intraciliary trafficking [50]. No association of *CCDC40, DNAH9*, *DNAH11*, *PIH1D3*, and *SPAG1* gene function with the immunological synapse has been identified so far. Moreover, all five genes are highly expressed in the respiratory system, primarily suggesting a role in respiratory epithelial function [47,51,52,53]. Within the heart, the ciliary function is discussed in endothelial regulation influencing hypertension and atherosclerosis [54]. Further, an accumulation of ciliated fibroblasts after myocardial injury could be detected indicating a potential role in the development of cardiac fibrosis [55]. Together, this raises the hypothesis that altered cilium function directly or indirectly predisposes for myocardial and/or inflammation in the context of CMP.

### Study Limitations

The interpretation of immune disease gene variants is limited due to the incomplete understanding of the clinical impact after monoallelic deactivation; a significant proportion of immune diseases are inherited recessively. Our genetic analysis approach assessed small genetic variation within or close to coding regions but did not test for other genomic alterations such as deep intronic or copy number variants. Currently, most of the exonic missense variants in immune disease genes are not interpretable due to a lack of knowledge. The study has an explorative character and requires complementation with further families and genetic burden analysis to solicit or reject the findings from the current analysis.

## 5. Conclusions

This study supports the idea that pediatric myocarditis with the DCM phenotype may be caused by the mutation of known CMP genes. Piloting the idea that additional immune-related genetic defects promote inflammation revealed truncation and (L)P variants in immune disease genes. These variants were present in the monoallelic, heterozygous state. Expanded analysis of individuals with pediatric myocarditis and the DCM phenotype will identify immune-related genetic factors predisposing one to myocardial inflammation.

## Figures and Tables

**Table 1:** Patient characteristics.

	n = 12
**General information**	
Male	5 (42)
Age (years)	1.6 (0.8–8.0)
BSA (m^2^)	0.5 (0.3–0.9)
**Symptoms**	
NYHA	
I	0 (0)
II	1 (8)
III	1 (8)
IV	10 (84)
Angina pectoris	1 (8)
Decompensation	12 (100)
Gastrointestinal symptoms	6 (50)
Infection (<6 weeks)	7 (58)
Fever (<6 weeks)	3 (25)
**ECG**	
ST-elevation	0 (0)
T-inversion	8 (67)
Arrhythmias *	5 (42)
**Laboratory**	
NT-proBNP (pg/mL) (N = 8)	23.025 (10.447–39.612)
Troponin elevated (N = 9)	6 (67)
**Echocardiography**	
Z-score LVIDd	6.6 (5.4–8.0)
LVEF (%)	23 (21–30)
**Endomyocardial Biopsy**	
Myocardial virus detection	6 (50)
*Diagnosis EMB*	
Acute myocarditis	1 (8)
Chronic healing myocarditis	8 (67)
Unspecific macrophages dominated inflammation	1 (8)
Unspecific macrophages dominated inflammation & DCM	2 (17)
**Medical treatment**	
Heart failure medication	13 (100)
Inotropic medication	13 (100)
Immunoglobulin	5 (42)
Valganciclovir/Ganciclovir	1 (8)
Azathioprine/Prednisolone	0 (0)
**Devices**	
ICD	1 (8)
Pacemaker	0 (0)
VAD	10 (83)
ECMO	0 (0)
Weaned overall (N = 11)	1 (8)
**Complications**	
Resuscitation	3 (25)
HTx	8 (67)
Death	0 (0)

Values are given as *n* (%) or median (interquartile range). * Arrhythmias were recorded with ECG and/or Holter-ECG and contained SVT, nsVT, VT. BSA = body surface area; DCM = dilated cardiomyopathy; ECG = Electrocardiogram; ECMO = extracorporal membrane oxygenation; EMB = endomyocardial biopsy; HTx = heart transplantation; ICD = implantable cardioverter-defibrillator; LVEF = left ventricular ejection fraction; LVIDd = left ventricular internal dimension at end-diastole; nsVT = non-sustained ventricular tachycardia; NT-proBNP = N-terminal pro brain natriuretic peptide; NYHA = New York Heart Association; SVT = supraventricular tachycardia; VAD = ventricular assist device; VT = ventricular tachycardia.

**Table 2:** Genetic CMP and immune disease variants.

Patient	Gene	Gene Class	Transcript	cDNA Position	Protein Position	Geno-Type	Consequence	ClinVar Annotation	Frequency GnomAD (Exomes)	CADD Value	ACMG Evaluation
Family #1	*TTN* ^AD^	CMP	ENSG00000155657	c.66547C > T	p.R22183*	het	stop gain	VCV000223326 (2LP, 4P)	0.0000040	67.0	LP
	*IRF7* ^AR^	IMMUNE	ENSG00000185507	c.21G > A	p.=	het	splice region	no	0	5.8	VUS
	*CCDC40* ^AR^	IMMUNE	ENSG00000141519	c.31_32insAAGCCGGGCCGGT	p.S11*	het	stop gain	no	0.0000308	21.9	VUS
Family #2	*TNNC1* ^AD^	CMP	ENSG00000114854	c.100G > A	p.G34S	het	*de novo*, missense	no	0	25.6	LP
	*FBN1* ^AD^	CMP	ENSG00000166147	c.6416A > G	p.H2139R	het	missense	VCV000200082 (4VUS)	0.000008	23.1	VUS
	*DMD* ^XLD,XLR^	CMP	ENSG00000198947	c.767A > G	p.Q256R	hemi	missense	no	0.000066	23.6	VUS
	*DNAH11* ^AR^	IMMUNE	ENSG00000105877	c.6151C > T	p.R2051*	het	stop gain	VCV000454692 (1P)	0.000017	40.0	LP (het)
Family #3	*TNNI3* ^AD^	CMP	ENSG00000129991	c.146T > A	p.L49Q	hom	missense	no	0	29.2	LP
	*SPAG1* ^AR^	IMMUNE	ENSG00000104450	c.2014C > T	p.Q672*	het	stop gain	VCV000088683 (1LP, 5P)	0.000082	38.0	LP (het)
	*FANCM* ^AR^	IMMUNE	ENSG00000187790	c.4223-4A > G	p.?	het	splice region	VCV001104556 (1VUS, 1LB)	0.000058	8.3	VUS
Family #4	*MYH7* ^AD^	CMP	ENSG00000092054	c.644C > T	p.T215I	het	*de novo*, missense	VCV000837897 (1VUS)	0	24.8	LP
Family #5	*MYLK2* ^AD^	CMP	ENSG00000101306	c.266G > T	p.G89V	het	missense	no	0	23.4	VUS
	*SGCG* ^AR^	CMP	ENSG00000102683	c.631A > G	p.I211V	het	missense	no	0	0.17	VUS
Family #6	*DNAH9* ^AR^	IMMUNE	ENSG00000007174	c.10479C > T	p.=	het	splice region	no	0.000079	6.5	VUS
Family #7	*TNNI3* ^AD^	CMP	ENSG00000129991	c.544G > C	p.E182Q	het	*de novo*, missense	no	0	23.9	LP
Family #8	*MYH7* ^AD^	CMP	ENSG00000092054	c.1633G > A	p.D545N	het	missense	VCV000264607 (2VUS, 1LP, 3P)	0	26.1	LP
	*MYH7* ^AD^	CMP	ENSG00000092054	c.2863G > A	p.D955N	het	missense	VCV000264608 (2VUS, 1LP, 3P)	0	31.0	VUS
	*ATRX* ^XLD,XLR^	IMMUNE	ENSG00000085224	c.6871A > G	p.I2291V	hemi	missense	VCV000210499 (1B, 3LB, 1VUS)	0	16.4	VUS
	*PIH1D3* ^XLR^	IMMUNE	ENSG00000080572	c.333G > A	p.=	hemi	splice region	no	0	6.9	VUS
Family #9	*TTN* ^AD^	CMP	ENSG00000155657	c.24597C > A	p.Y8199*	het	stop gain	no	0	44.0	LP
	*FANCC* ^AR^	IMMUNE	ENSG00000158169	c.349_360del	p.V117_H120del	het	inframe deletion	VCV000970500 (2VUS)	0	19.2	VUS
Family #10 [44]	*FKTN* ^AR^	CMP	ENSG00000106692	c.895A > C	p.S299R	het	missense	VCV000264590 (2VUS)	0.000008	29.1	VUS
	*FKTN* ^AR^	CMP	ENSG00000106692	c.1325A > G	p.N442S	het	missense	VCV001022112 (1VUS)	0.000004	25.2	VUS
	*FANCC* ^AR^	IMMUNE	ENSG00000158169	c.1642C > T	p.R548*	het	stop gain	VCV000012047 (1LP, 13P)	0.000025	36.0	LP (het)
	*FLNA* ^XLR;XLD^	IMMUNE	ENSG00000196924	c.49C > G	p.P17A	hemi	missense	VCV000393067 (1VUS)	0.000014	15.9	VUS
	*RAC2* ^AD,AR^	IMMUNE	ENSG00000128340	c.*2 + 1del	p.=	het	splice region	no	0	31	VUS
Family #11	*RYR2* ^AD^	CMP	ENSG00000198626	c.3265G > A	p.E1089K	het	missense	VCV000180493 (1VUS, 1LP)	0.000008	27.2	LP
	*PLG* ^AD,AR^	IMMUNE	ENSG00000122194	c.1675C > T	p.Q559*	het	stop gain	no	0	36.0	VUS
Family #12	*SDHA* ^AD,AR^	CMP	ENSG00000073578	c.1951G > A	p.E651K	het	missense	VCV000220782 (2VUS)	0.000019	21.9	VUS
	*DSC2* ^AD,AR^	CMP	ENSG00000134755	c.1309G > C	p.V437L	het	missense	VCV000925401 (1VUS)	0	22.8	VUS
	*RBCK1* ^AR^	IMMUNE	ENSG00000125826	c.583_584dup	p.A196Efs* 39	het	stop gain	no	0	32.0	VUS

Abbreviations: ATRX—ATRX chromatin remodeler (XLD, XLR); CCDC40—coiled-coil domain containing 40 (AR); DMD—dystrophin (XL, XLR); DNAH9—dynein axonemal heavy chain 9 (AR); DNAH11—dynein axonemal heavy chain 11 (AR); DSC2—desmocollin 2 (AD, AR); FANCC—FA complementation group C (AR); FANCM—FA complementation group M (AR); FBN1—fibrillin 1 (AD); FKTN—fukutin (AR); FLNA—filamin A (XLD, XLR); IRF7—interferon regulatory factor 7 (AR); MYH7—myosin heavy chain 7 (AD); MYLK2—myosin light chain kinase 2 (AD); PLG—plasminogen (AD, AR); PIH1D3—PIH1 domain-containing protein 3 (XLR; novel gene name DNAAF6); RAC2—Rac family small GTPase 2 (AD, AR); RBCK1—RANBP2-Type And C3HC4-Type Zinc Finger Containing 1 (AR); RYR2—ryanodine receptor 2 (AD); SDHA—succinate dehydrogenase complex flavoprotein subunit A (AD, AR); SGCG—sarcoglycan gamma (AR); SPAG1—sperm associated antigen 1 (AR); TNNC1—troponin C1, slow skeletal and cardiac type (AD); TNNI3—troponin I3, cardiac type (AD); TTN—titin (AD); AD—autosomal dominant; AR—autosomal recessive; het—heterozygous; hom—homozygous; LP—likely pathogenic; P—pathogenic; VUS—variant of unknown significance; XLD—X-linked dominant; XLR—X-linked recessive; CADD—Combined Annotation-Dependent Depletion score.

## Data Availability

Not applicable.
